# All-dielectric metasurfaces with high Q-factor Fano resonances enabling multi-scenario sensing

**DOI:** 10.1515/nanoph-2022-0394

**Published:** 2022-09-19

**Authors:** Xueer Chen, Yong Zhang, Guoxiong Cai, Jianliang Zhuo, Kunzhong Lai, Longfang Ye

**Affiliations:** Institute of Electromagnetics and Acoustics, School of Electronic Science and Engineering, Xiamen University, Xiamen 361005, China; Shenzhen Research Institute of Xiamen University, Shenzhen 518057, China; School of Electronic Science and Engineering, University of Electronic Science and Technology of China, Chengdu 611732, China

**Keywords:** all-dielectric metasurface, bound states in the continuum (BIC), sensing

## Abstract

We propose and numerically demonstrate high Q-factor sensors based on all-dielectric metasurfaces, which are very sensitive to the change of the refractive index of the surrounding media and the incident angle. By using the light incident angular scanning method, the all-dielectric metasurface based on symmetric tetramer can act as an excellent sensing platform for trace-amount molecules such as protein A/G, 2, 4-DNT, and 2D material graphene with huge absorbance enhancement in the mid-infrared broadband spectrums. The results reveal that envelope of absorbance amplitudes is in good agreement with the vibrational mode of molecules, and absorbance enhancement factors reach as high as 10 dB in the mid-infrared wavelength range from 5.75 to 6.80 μm. To further increase the Q-factor of the resonances, the all-dielectric metasurface based on asymmetric tetramer is investigated. This asymmetric structure can induce toroidal and magnetic dipoles governed by quasi-BIC to produce multi-extremely narrow linewidth Fano resonances, and the maximum sensitivity reaches up to 1.43 μm/RIU. Therefore, the proposed all-dielectric metasurface demonstrates highly enhanced performance in refractive index and chemical information sensing for trace-amount biomolecules, which inspires the development of new high-sensitivity refractive index sensors for the nondestructive identification in the mid-infrared regime.

## 1 Introduction

Optical sensing is a powerful technique to identify analytes in a wide range of fields including biochemical detection, environmental toxic monitoring, and security inspection. Nondestructive and label-free optical sensing with the ability to measure molecular concentrations and interaction kinetics without interference from fluorescent labels or other external tags [1] has attracted increasing interest in the detection and distinguishing target analytes like proteins, lipids, and DNA [2]. However, the sensitivity for nanometer-scale and trance-amount analytes is very limited owing to the mismatch between wavelengths and molecule sizes [2]. Metasurface, composed of multiple subwavelength nanoresonators, can be used to manipulate electromagnetic waves in the microwave, terahertz, and optical regions. In recent years, many optical devices based on metasurface have been proposed including absorbers [3, 4], optical modulators [5, 6], polarization converters [7, 8], optical refractometric sensors [9, 10], etc. Mertasuface is a powerful optical sensing platform with increased sensitivity. It can confine light into nanoscale electromagnetic hotspots with strong near-field enhancement by proper design. Based on the metal nanoparticle array, the plasmonic metasurfaces have been applied in chemical detection and biosensing [11, 12]. However, owing to the intrinsic losses of the constituent metals, the plasmonic metasurface suffers from inherent low-Q (quality) factor resonance limitations.

All-dielectric metasurfaces are usually composed of structured high-index dielectric materials, which have emerged in various optical sensing applications. Compared with plasmonic metasurfaces, they have the advantages of smaller dissipation, lower thermal conductivity, and also much stronger magnetic resonances. All-dielectric metasurfaces have drawn surging attention because of their great potential for high-efficiency ultrasensitivity sensing. For example, the strong Mie-type [13] resonances supported by high-index dielectric metasurfaces produce near-field hotspots, which could enhance the light–matter interactions for the analytes around the hotspots of the resonators. In the last few years, many all-dielectric metasurface sensors have been demonstrated for label-free optical sensing. Generally, the sensing approaches can be classified into refractometric detection, surface-enhanced spectroscopy, as well as chiral molecular sensing [14]. For refractometric detection, the all-dielectric metasurface is coated with analytes. The surrounding media binds to the resonators of the metasurface and creates local changes in the refractive index, leading to the resonance frequency shift for the molecule identification. While, the surface-enhanced Raman spectroscopy (SERS) [15, 16] and surface-enhanced infrared absorption spectroscopy (SEIRAS) [17, 18] are the other methods for vibrational spectroscopy and biomolecule sensing, which not only allow for detecting surface-bound molecules but also provides chemically specific information about each analyte. Recently, Tittl, et al. proposed a pixelated dielectric metasurface [2] for biosensing and environmental monitoring. They read out absorption signatures of biomolecules, polymers, and pesticides by broadband molecular fingerprint sensing [19] and translated the information into a barcode-like spatial absorption map for imaging. Broadband molecular fingerprint sensing is a nondestructive and label-free chemically optical method. Intrinsic vibrational modes of molecular chemical bonds lead to characteristic absorption under mid-infrared optics [14]. However, for some tiny surface-bound molecule target analytes or ultra-thin trace substances, the spectroscopy influence is usually limited and the differential readout of vibrational information before and after analyte coating is small. All-dielectric metasurfaces with high Q-factor resonances are proposed to improve the sensitivity. Leitis et al. proposed an all-dielectric metasurface with ellipse-shaped resonators [19] to improve the sensing signatures. They experimentally demonstrated that all-dielectric metasurface can act as a high-efficiency angle-scanning refractometric sensor using broadband light sources and detectors. Besides, metasurface combined with incident light angle, resonator size, or thickness multiplexed methods may provide an efficient tool for extracting broadband molecular fingerprints.

In this paper, we numerically demonstrate a variety of silicon square tetramer dielectric metasurfaces with optimized sensing performance and their applications in both refractometric detection and broadband fingerprint sensing for trace-amount substances. Compared to the previously reported refractometric sensors [20–22], the proposed metasurface can provide multi-Fano resonances with a higher Q-factor of 1.71 × 10^5^, and a simpler geometry design without the need for a complicated fabrication process. Compared to the previously proposed biomolecule sensing [23], it can achieve a broader wavelength coverage ranging from 5.75 to 6.80 μm and a higher broadband absorbance enhancement reaching 10 dB. The proposed metasurface is a high-performance sensing platform for different kinds of analytes such as ultrathin monolayer graphene and trance-amount substances, where the retrieving angle-scan signals are correlated well with the molecular vibrational mode and chemically specific information. Moreover, the proposed silicon-based metasurface enables strong light confinement and reduces heat generation, and therefore, it could somehow avoid biomolecule damage and local refractive index change, or even deform the metasurface itself [24, 25]. By combining the above two sensing methods with the same simple design, the proposed metasurface not only tracks resonance frequency shift but also extracts chemically specific information from various analytes, which may facilitate more label-free and nondestructive trace-amount detecting scenarios.

## 2 Symmetric tetramer metasurface with single Fano resonance

In this work, we simulate the all-dielectric metasurface unit cell by assigning the periodic boundaries in both *x*- and *y*-directions, and Floquet ports in the *z*-direction under transverse electric (TE) polarization. The absorbance is calculated by *A* = 1 − *R* − *T*, where the reflectance *R* = |*S*_11_|^2^ and the transmittance *T* = |*S*_21_|^2^. Since the proposed unit cell possesses polarized symmetry, the reflectance, transmission, and absorbance of the modulator under TE and TM polarizations should be the same, and therefore, we only focus on the performance discussion under TE polarized incidence.

### 2.1 Design, results and discussion

**Figure 1:**
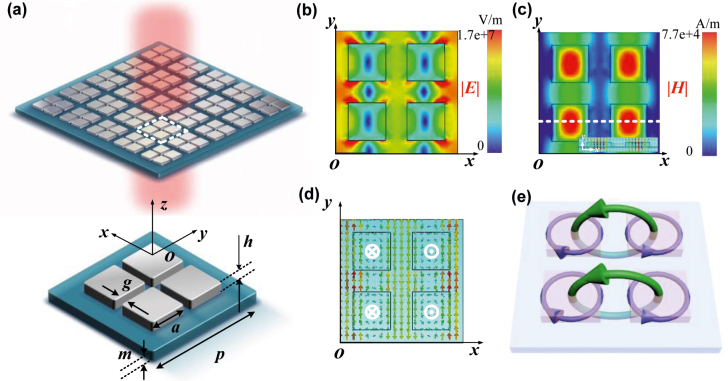
Schematic diagram of the proposed symmetric metasurface. (a) 3D schematic diagram of the symmetric metasurface, where *p* = 6.2, *m* = 0.36, *g* = 1.1, *a* = 1.9, *h* = 0.3 μm. (b) Electric field profile in *x*–*y* mid-plane. (c) Magnetic field profile in *x*–*y* plane and magnetic field vector in *x*–*z* plane of Fano resonance. (d) Electric field vector profile. (e) 3D schematic diagram of dual toroidal dipoles in the tetramer.

The schematic diagram of the proposed symmetric tetramer metasurface is depicted in Figure 1(a). The metasurface consists of four identical Si square nanodisks (*ε*_si_ = 11.71) placed on a SiO_2_ substrate (*ε*_sio2_ = 1.79). The geometric parameters of the unit cell are set as follows, *p* is the period width along the *x* and *y* axes, *m* is the thickness of the SiO_2_ substrate, *a* and *h* are the side length and thickness of the four Si square nanodisks, and *g* is the gap between adjacent square nanodisks. In this design, owing to the strong coupling among adjacent nanodisks supported by a certain dipole, a sharp BIC resonance is excited in the mid-IR range. Fano resonance is a kind of asymmetric lineshape of a spectrum phenomenon, which is attributed to the interference of a continuum (or broad) state and a discrete (or narrow) state [26]. The sharp Fano resonances of the proposed metasurface are desirable in refractive index sensing. To intuitively investigate the resonance mechanism, we calculate the electric and magnetic field distributions of the unit cell at the resonance wavelength of 6.87 μm in Figure 1(b) and (c), respectively. The electric fields of this resonance are mainly located in the gap of the square disks and the corresponding electric field vectors are shown in Figure 1(d). Notably, the direction of the electric field vectors in the adjacent square disks are twisting in the opposite directions and produces four longitudinal magnetic moments (white crosses and dots in Figure 1(d)) oriented along the *z*-axis inversely located in the center of the square disks. The direction arrows of the magnetic fields with head-to-tail links in two pairs form dual parallel magnetic toroidal dipoles [27, 28]. Figure 1(e) shows a 3D schematic diagram of the supporting dual parallel magnetic toroidal dipoles (MTD) (the green circular column), which are created by the circular displacement currents (the purple circular column) in the *x*–*y* plane. As we know, there are two primitive toroidal dipoles [29]: one is the magnetic toroidal dipole (MTD) and the other is the electric toroidal dipole (ETD). The MTD is created by poloidal electric currents flowing on a surface of a torus along its meridians, and it can also be viewed as a set of magnetic dipoles arranged head-to-tail to form a closed loop. And the ETD is composed of a circular closed loop of electric dipoles. The mode inside of the particle clusters is an intracluster toroidal dipole; the mode between the neighboring particles in the clusters is an intercluster toroidal dipole. For this symmetric tetramer structure, Fano resonance is produced and supported by dual magnetic toroidal dipole (MTD) of intracluster.

**Figure 2:**
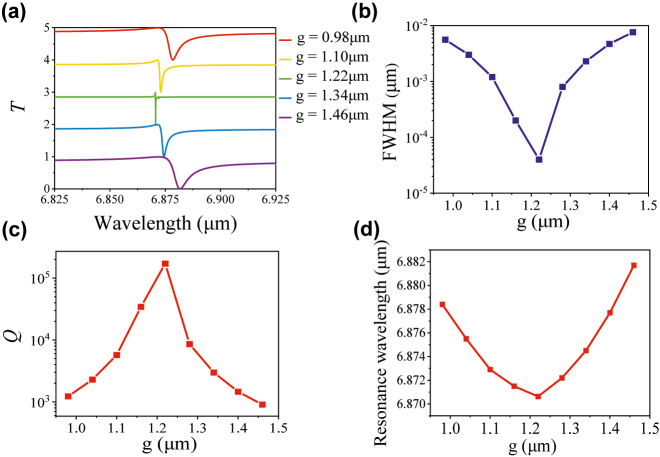
Simulated transmission spectra of the symmetric metasurface. (a) The transmission spectra. (b), (c) and (d) are FWHM, Q-factor, and resonance wavelength under different gap distance *g* between adjacent square disks, respectively.

Next, to study the Fano resonance performance with different geometry parameters and further optimize its Q-factor, we explore the effect of the gap *g* of adjacent square nanodisk on the Fano resonance wavelengths and full width at half-maximum (FWHM) of the transmission spectra of the proposed symmetric tetramer unit cell. As shown in Figure 2(a), the transmission spectra at different gap sizes are presented. It is found that the resonant wavelength has a blue-shift when the gap *g* increases from 0.98 to 1.22 μm, while has a red-shift when the gap *g* further increases from 1.22 to 1.46 μm*.* The extracted Q-factor, FWHM, and resonance wavelength as the criteria for sensor properties are shown in Figure 2(b)–(d), respectively. Clearly, the minimum FWHM of Fano resonance is 4 × 10^−5^ μm, and the corresponding high Q-factor reaches up to 1.71 × 10^5^ when *g* = 1.22 μm, showing great potential in ultrasensitive biosensor applications.

**Figure 3:**
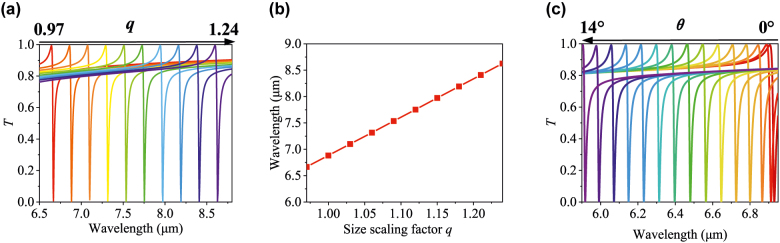
The dependence of the Fano resonance wavelengths on the scaling factor *q *and incident angle *θ*. (a) Transmission spectra of the metasurface with different square disk size scaling factor *q*, where other parameters remain as the original settings. (b) The linear relation between resonant wavelength and *q*. (c) Transmission spectra with different incident angles.

Before the discussion of broadband sensing performance, we study the dependence of the Fano resonance wavelength of the proposed symmetric tetramer metasurface on the scaling factor *q* for the size length of the Si nanodisks (*a*′ *= a* × *q*) and incident angle *θ*. Aiming to use the bandwidth efficiently, the Fano resonant wavelength is moved to a specific range via tuning the scaling factor *q.* As shown in Figure 3(a) and (b), all the transmission spectra under different scaling factors *q* have very similar Fano resonances and the Fano resonance wavelength increases linearly from 6.67 to 8.63 μm as *q* increases 0.97–1.24. Therefore, without the need for a complicated structure redesign, we can flexibly tune the Fano resonance wavelength to the certain targeted range only by adjusting scaling factor *q* for Si nanodisks of the metasurface, which is very convenient for various sensing applications in different wavelength ranges. Next, to use the angle-multiplexed method to read out a series of transmission and absorption signatures of analytes for broadband molecular fingerprint sensing [19], the proposed sensor should be dependent on the incident angle. Figure 3(c) demonstrates that the resonance wavelength of the transmission spectra is sensitive to incident angle *θ*, which is of great significance for broadband sensing.

### 2.2 Sensing performance on detection of materials with complex refractive index

Metasurface combined with the angle-multiplexed method can act as an efficient platform for broadband molecular fingerprint sensing. In the process of detection, we utilize the linear relationship between high Q-factor Fano resonance wavelength dip of the transmission spectrum as a function of the incident angle (see Figure 3(c)) to retrieve a series of broadband fingerprint spectra. The broadband fingerprint spectra are related to the chemical characteristics of coated analytes. Owing to the high Q-factor resonance, giant absorbance enhancement is achieved. In this section, we use the angle-multiplexed method for the detection of several ultrathin analytes with complex refractive indices, such as protein A/G biomolecules, single-layer graphene, and explosive 2, 4-dinitrotoluene. Considering the linear relationship between the Fano resonance wavelength and size scaling factor *q* (see Figure 3), we choose size scaling factor *q* as 0.974, 1.23, and 1.1 for the metasurface design for the detection of protein A/G biomolecules with a high extinction coefficient in 5.25–7.75 μm, single-layer graphene in 7.3–8.8 μm, and explosive in 6–7 μm, respectively.

#### 2.2.1 Detection of biomolecules

**Figure 4:**
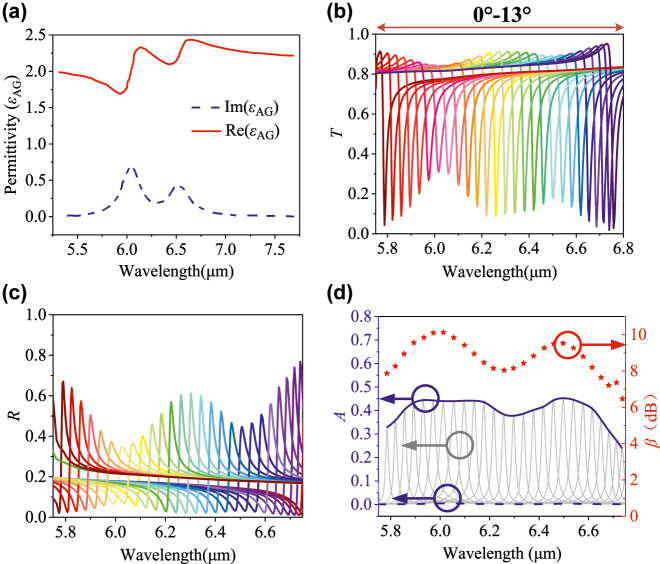
Permittivity of protein A/G and its detection results. (a) Real part and imaginary part of permittivity of protein A/G. (b) and (c) are transmission and reflection spectra under incident light from 0° to 13° with a step size of 0.5°. (d) Absorbance spectra and enhancement factor of proposed metasurface sensor with protein A/G film.

Firstly, we demonstrate molecular fingerprint sensing of recombinant protein A/G biomolecules [19]. The real and imaginary parts of the protein A/G permittivity are shown in Figure 4(a). It has two optical loss peaks located at amide I and II around 6.1 and 6.6 μm, respectively. In the simulation, the proposed metasurface is coated with the 8 nm ultra-thin protein biomolecule film under different incident angles ranging from 0 to 13° (the red line). As shown in Figure 4(b) and (c), the envelopes of transmission dips and reflection peaks are corresponding to the vibrational mode of the protein A/G molecules. Then, we extract the envelope of the absorbance spectra peak of biomolecule film on the proposed metasurface and compare it with that on a flat SiO_2_ substrate (the blue dashed line) in Figure 4(d). It is found that even the biomolecule film is as thin as 8 nm, the peak absorbance with the proposed metasurface reaches up to 44.8%, which is much larger than near to zero absorbance with a flat SiO_2_ substrate. The sensing performance via metasurface shows obvious infrared absorbance enhancement compared to the sensing signatures without metasurface. To quantify absorbance enhancement of the proposed metasurface, we calculate broadband enhancing factors of absorbance *β* [23] from the absorbance amplitudes *A* at each incidence angle by the formula: $\beta ={10\text{l}}_{\text{g}}\left(\int_{\lambda_{1}}^{\lambda_{2}}A_{\text{metasurface}}\text{d}\lambda /\int_{\lambda_{1}}^{\lambda_{2}}A_{\text{substrate}}\text{d}\lambda \right)$ , where *A*_metasurface_ and *A*_substrate_ are the wavelength-dependent absorbance of the metasurface and the flat substrate after coating biomolecules, respectively. Figure 4(d) shows that the maximum enhancement factor of protein A/G is 10.12 dB. It is clear that the enhancement envelopes are consistent with the corresponding biomolecule vibrational modes.

#### 2.2.2 Detection of two-dimensional material

**Figure 5:**
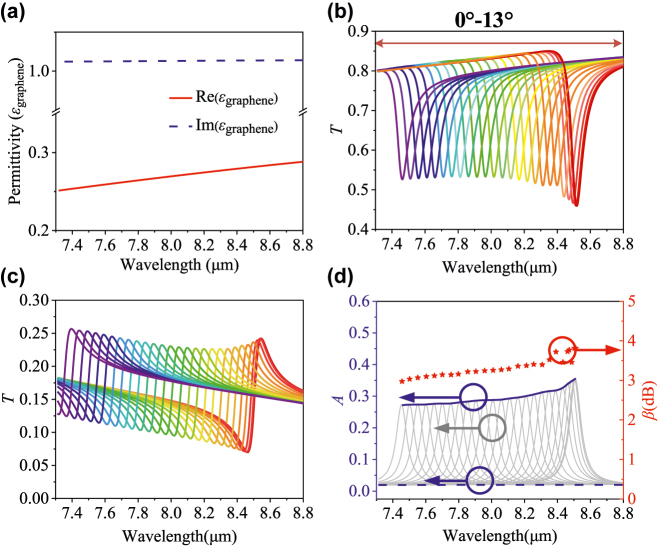
Permittivity of graphene and its detection results. (a) Real part and imaginary part of permittivity of graphene monolayer. (b) and (c) are transmission and reflection spectra under incident light from 0° to 13° with a step size of 0.5°. (d) Absorbance spectra and enhancement factors of proposed metasurface with graphene film.

The proposed metasurface not only can be used to detect biomolecules but also can be used to identify 2D materials. As one of the most common 2D materials, graphene has been applied widely in various optical devices [30]. The thickness of graphene monolayer is only 0.34 nm, and its high transmission makes it hard to be identified only by human eyes without any auxiliary equipment such as scanning electron microscopy or Raman spectrometer. Because of the enhanced localized fields, the proposed metasurface can be used to detect coating graphene monolayer. Figure 5(a) shows the permittivity of graphene in the wavelength range of 7.3–8.8 μm. Then, we adjust the scaling factor *q* as 1.23 of the side length *a* of the metasurface to match this target wavelength range. The simulated *T*, *R*, and *A* of the metasurface coated with single graphene under different incident angles ranging from 0 to 13° are shown in Figure 5(b)–(d), respectively. The extracted absorbance peak envelope (the red curve of Figure 5(d)) reproduces the graphene extinction coefficient trend in Figure 5(a). Compared to the maximum absorption of 1.93% of graphene on an unpatterned substrate (the blue dashed curve), the proposed metasurface can achieve significant absorbance enhancement from 26.8 to 34.2%, the corresponding enhancement factor from 2.97 to 3.81 dB in 7.3–88 μm. Therefore, the proposed metasurface possesses excellent ultrathin analytes identification performance, which may have potential applications in the detection of diverse 2D materials such as silicone, h-GaN, h-SiC, h-BN, and h-BAs.

#### 2.2.3 Detection of 2, 4-DNT

**Figure 6:**
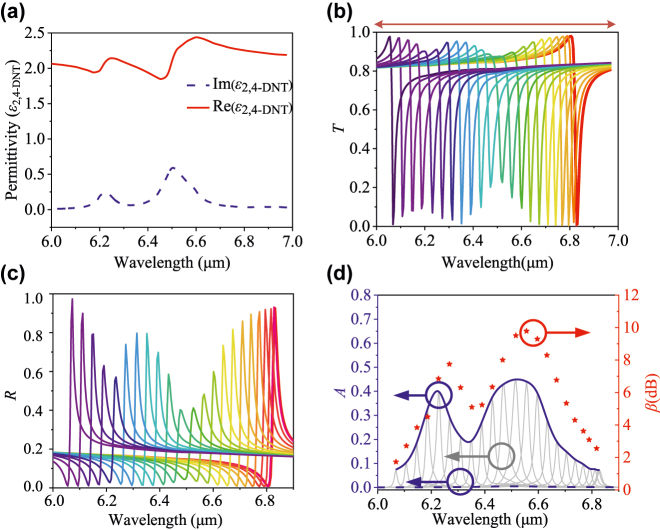
Permittivity of 2, 4-DNT and its detection results. (a) Real part and imaginary part of permittivity of 2, 4-DNT (b) and (c) are transmission and reflection spectra under incident light from 0° to 11° with a step size of 0.5°. (d) Absorbance spectra and enhancement factors of proposed metasurface sensor with 2, 4-DNT overlayer.

Furthermore, we investigate the broadband fingerprint sensing of trace-amount explosive 2, 4-dinitrotoluene (DNT). 2, 4-DNT is a precursor of the widespread explosive 2, 4, 6-trinitrotoluene (TNT) [31]. The detection of traces of 2, 4-DNT indicates the presence of TNT. Figure 6(a) provides the real and imaginary parts of the 2, 4-DNT permittivity, which has two obvious extinction coefficient peaks around 6.22 and 6.51 μm. To achieve complete spectral coverage, we set the size scaling factor *q* to 1.1. The simulated *T*, *R*, and *A* of the metasurface deposited with an 8 nm 2, 4-DNT thin layer under different incident angles ranging from 0 to 13° are shown in Figure 6(b)–(d), respectively. It is found that the envelope of absorbance peaks of the metasurface with 2, 4-DNT (red curve in Figure 6(d)) is in good agreement with the imaginary part of permittivity shown in Figure 6(a), and the absorbance enhancement factor of 2, 4-DNT with metasurface can reach as high as 9.78 dB at 6.56 μm.

The above results indicate the proposed metasurface is a promising high-sensitivity sensing platform, which can be used to strengthen the weak fingerprint absorbance of various ultrathin trance amount analytes in a wide range of wavelengths. Besides, the absorbance enhancement factor of the metasurface mainly depends on the refractive index, thickness of analyte, and the near-field distributions, leading to the different values of enhancement factors for different analytes, as shown in Figures 4(d)– 6(d). In addition, the Q-factor of the metasurface can be further improved by tuning the gap between the resonator without complicated structure redesign and sophisticated nanofabrication. Although a narrower FWHM and a higher Q-factor bring better sensitivity and resolution, they may result in broad spectral coverage limitation and sampling inaccuracy problems. Therefore, it needs tradeoff between high absorbance enhancement and broad bandwidth coverage in the metasurface design for broadband fingerprint detection applications.

## 3 Asymmetric tetramer metasurface with multi-Fano resonances

### 3.1 Design, results and discussion

**Figure 7:**
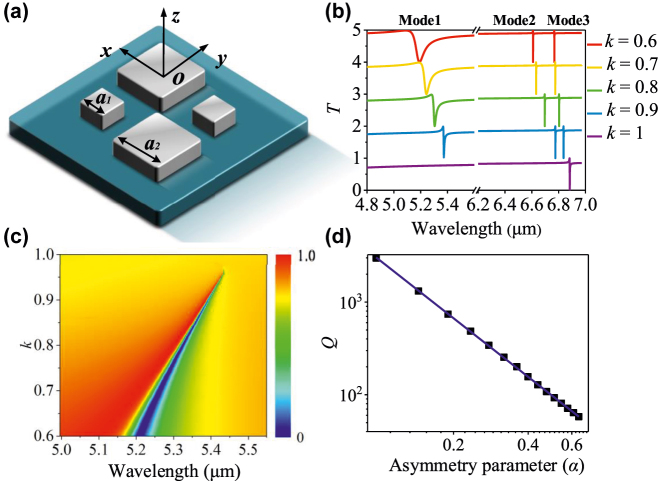
Schematic configuration and multi-Fano resonance characteristics of the proposed asymmetric metasurface. (a) 3D schematic diagram of the asymmetric metasurface. (b) The transmission spectra under different linearly scaled factor *k.* (c) The transmission spectra of Mode 1 under different linearly scaled factor *k.* (d) Q-factors extracted from calculated spectra under different asymmetric parameters of Mode 1.

To further increase the Q-factor of the resonances and enrich detection signals, we transformed the symmetry-protected BIC [32] tetramer metasurface design into a quasi-BIC state by breaking the symmetry of the unit cell as shown in Figure 7(a). The quasi-BIC can induce resonant interference between a collective subradiant dark mode and the superradiant collective bright mode, and therefore, the asymmetric dielectric tetramer metasurface can support multi-Fano resonances in mid-infrared. To make it insensitive to the polarization of the incident light, we arrange two pairs of nanodisks in the diagonal direction of the unit cell. The transmission spectra as a function of different linearly scaled factor *k* (*k* = *a*_1_/*a*_2_) ranging from 0.6 to 1 are shown in Figure 7(b) and (c), where the side length of the larger square nanodisk and the smaller nanodisk are set as *a*_1_ = 1.9 and *a*_2_ = 1.9/*k* μm, respectively. Clearly, for *k* = 1 (symmetric structure), only one Fano resonance (Mode 3) can be observed at 6.87 μm. The symmetry is broken when the scaled factor *k* ≠ 1, leading to the energy excitation of additional Fano resonance produced by the interference between discrete states supported by metasurface and the continuum free space radiation [33]. Clearly, as shown in Figure 7(b), the transmission spectra show two additional Fano resonances (Mode 1 and Mode 2) when *k* = 0.6, 07, 0.8, and 0.9. As *k* increases, the Mode 1 resonance will show a redshift, a narrower FWHM, and a higher Q-factor.

We further investigate the relationship between the Q-factor of Mode 1 and the asymmetry parameter *α* of the metasurface, where *α* = Δ*S*/*S* = (*a*_2_^2^ − *a*_1_^2^)/*a*_2_^2^ = 1 − *k* ^2^. According to previous research reported by Koshelev *et al*. [32], the relationship of the radiative Q-factor of quasi-BIC on the asymmetry parameter meets the inverse quadratic law (*Q*_rad_ *∝ α* ^−2^). As shown in Figure 7(d), the relation between the Q-factor of Mode 1 and the asymmetry parameter is consistent with the inverse quadratic law, which indicates the resonance is excited by quasi-BIC. As *α* increases from 0.64 to 0.09, the Q-factor will significantly increase from 57.95 to 2985.80, which will be further improved by orders when the asymmetry parameter *α* is enough low.

**Figure 8:**
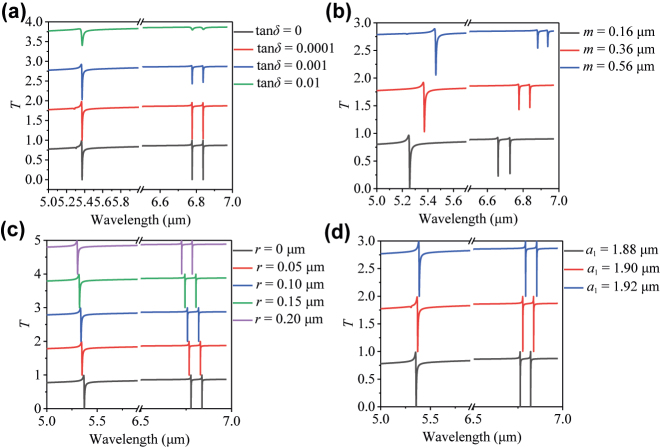
The influence of material loss and fabricated imperfection on transmission spectra. (a) Transmission spectra for different loss tangents of SiO_2_ substrate. (b) Transmission spectra for different SiO_2_ substrate thicknesses with tan*δ* = 0.001. (c) Transmission spectra for different radius *r* of rounded corners. (d) Transmission spectra for the different silicon square side length *a*_1_.

Then we study the impacts of the loss tangent and thickness of SiO_2_ substrate and the dimension imperfections of Si nanodisks on the resonance properties of the asymmetric tetramer metasurface. Considering the small loss of the SiO_2_ substrate [34] in the mid-IR, we simulate the influence of different loss tangents of the SiO_2_ substrate (tan*δ* = 0, 0.0001, 0.001, 0.01) on the metasurface spectra. As shown in Figure 8(a), it is observed that the large tan*δ* will deteriorate the Q-factor of the spectrum resonances to some extent. However, when tan*δ* is smaller than 0.001, the influence of SiO_2_ attenuation on transmission spectra is much smaller. The impact of substrate thickness on the transmission spectra is simulated and shown in Figure 8(b). Clearly, by reducing the substrate thickness, the FWHM becomes smaller and the Q-factor of resonance becomes higher with SiO_2_ tan*δ* = 0.001. This implies that the loss can be further minimized by decreasing the substrate thickness. Therefore, one good way to circumvent the SiO_2_ substrate loss is using enough thin substrates. For example, metasurface with freestanding membrane as the supporting layer could be a possible way to further reduce the loss from the substrate and improve the dielectric symmetry [35, 36]. Furthermore, the effects of sample imperfections on the resonance properties of the transmission spectra are also investigated. For example, we assume that the metasurface has round corner imperfections in the Si nanodisks. As shown in Figure 8(c), the large rounded corner radius *r* changes (from 0 to 0.2 μm) only induce a slight blue shift of the resonance, while remaining its FWHM almost unchanged. Moreover, the dependence of the transmission spectra on the silicon square size *a*_1_ ranging from 1.88 to 1.92 is shown in Figure 8(d). Similarly, the small *a*_1_ variation causes some frequency shifts, but the resonance line width almost remains the same. Therefore, small sample imperfections have a small impact on the sensing properties, showing high facilitation tolerance.

**Figure 9:**
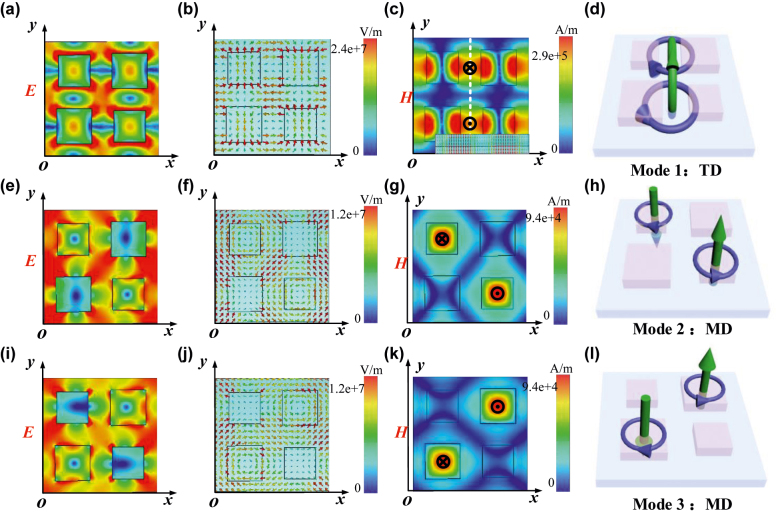
Electromagnetic field distributions and resonance modes. (a), (b), (c), and (d) are electric field, electric field vector, magnetic field profiles, and 3D schematic diagram of TD of Mode 1. (e), (f), (g), and (h) are electric field, electric field vector, magnetic field profiles, and 3D schematic diagram of TD of Mode 2. (i), (j), (k), and (l) are electric field, electric field vector, magnetic field profiles, and 3D schematic diagram of TD of Mode 3.

To better understand the resonance mechanism, Figure 9(a) shows the distribution of electric and magnetic field amplitude of Mode 1. The two antiphase magnetic dipoles form a head-to-tail arrangement and further produce two MTD resonances oscillating reversely parallel to the surface of the metasurface along the *z*-direction. The Fano resonances of Mode 2 and Mode 3 have high Q-factors over 10^4^, and their resonance wavelength exhibits a blue shift as asymmetry parameter *α* increases. As shown in Figure 9(b) and (c), we display the electromagnetic field distributions of these modes. Clearly, Mode 2 is excited by dual inverted MDs diagonally, where two closed loops of the displacement currents appear in two relatively smaller nanodisks of four clusters and produce reversed magnetic field lines. Similarly, Mode 3 is also excited by dual inverted MDs diagonally, where the closed-loop of displacement currents appeared in two relatively larger nanodisks forming two antiphase MDs.

**Figure 10:**
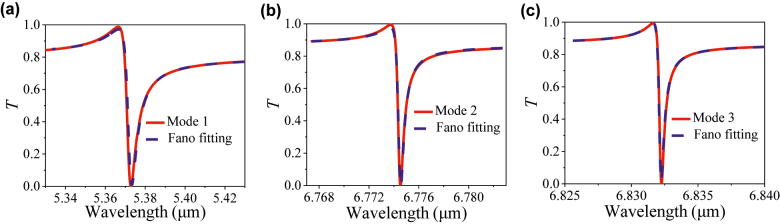
Comparison of the Fano fitting and simulation results. (a), (b), and (c) are Fano fittings of the three modes when *k* = 0.9. The solid curves are simulation results, and the dashed curves are Fano fitting results.

Furthermore, to gain insight into the three asymmetrical lineshapes formation of Fano resonances of Modes 1, 2, and 3, we fit transmission spectra with reproduced Fano model by using the classical Fano formula [21] $T\left(\omega \right)=T_{0}+A_{0}\frac{\left[q+2\left(\omega -\omega_{0}\right)/\tau \right]}{1+{\left[2\left(\omega -\omega \right)/\tau \right]}^{2}}$ , where *ω*_0_ is the resonant frequency, *τ* is the resonance FWHM, *T*_0_ is the transmission offset, *A*_0_ is the continuum-discrete coupling constant, *q* is the Breit–Wigner–Fano parameter determining asymmetry of the resonance profile. Figure 10 shows that the prediction of three Fano models (red solid curve) under *k* = 0.9 agrees well with the simulation results (blue dashed curve) around the resonance dips, indicating the Fano resonance essence. With the fitting results, Q-factors of Modes 1, 2, and 3 reach 818.72, 2.23 × 10^4^, and 1.34 × 10^4^, respectively.

### 3.2 Sensing performance on detection of liquid with various refractive index

**Figure 11:**
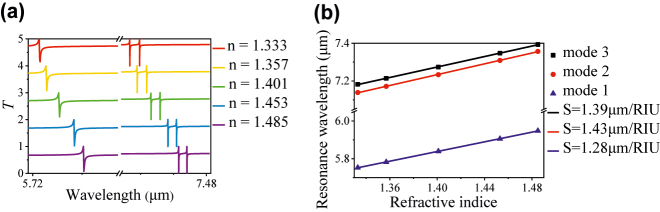
Refractive sensing performance of the asymmetric metasurface. (a) Transmission spectra of the metasurface with different refractive indices of analytes. (b) The resonant frequency shift of three Fano modes with different refractive indices of analytes.

For the dielectric tetramer metasurface with the asymmetric nanodisks, multi-Fano resonances with high Q-factor are excited, which provide excellent sensing properties to identify liquid with various refractive index *n*. As an example, here, we simulate the transmission T and evaluate the sensitivity of the asymmetric metasurface (*k* = 0.9) immersed by 0.6-μm thickness liquid analytes. The *T* spectra of different refractive indices of analytes such as water with *n* = 1.333, ethanol (C_2_H_5_OH) with *n* = 1.357, pentanol (C_5_H_11_OH) with *n* = 1.401, carbon tetrachloride (CCl_4_) with *n* = 1.453, and benzene (C_6_H_6_) with *n* = 1.485 are depicted as Figure 11(a). It is found that three Fano resonances exhibit a redshift with the increase of the analyte refractive index. Moreover, we use the sensitivity (*S* = Δ*λ*/Δ*n*), namely spectral shift per refractive index unit, to evaluate the sensing properties of the metasurface. As shown in Figure 11(b), the *S* of Modes 1, 2, and 3 is 1.28, 1.43, and 1.39 μm/RIU, respectively. In addition, Table 1 compares the performance of our proposed all-dielectric metasurface sensor with the recently reported refractive sensors in the infrared region. It is found that our structure shows obvious superior to previous work in terms of sensitivity, Q*-*factor*,* and polarization insensitivity, demonstrating good sensing performance.

**Table 1:** Comparison of the proposed refractive index sensors with recent work in the infrared region.

Year^Ref^	Structure	Material	*Q*	*S* (nm/RIU)	Polarization sensitivity
2016 [22]	Cylinder	InSb	1.4 × 10^4^		Insensitive
2017 [37]	Square-lattice circular PC holes	Si	–	500	–
2021 [38]	Slotted-waveguide coupled-cavity	SOI	1.0821 × 10^7^	1343.2	–
2021 [39]	Lucky knot	Si	520	986	Insensitive
2021 [40]	Square	Metal/dielectric/metal	–	667	Insensitive
This paper	Square	Si	818.72	1280	Insensitive
			2.23 × 10^4^	1430
			1.34 × 10^4^	1390

Finally, a possible large-scale metasurface sample fabrication process flow is discussed. The proposed metasurface can be fabricated by using the state-of-the-art nanofabrication techniques like nanoimprint, electron beam lithography (EBL), and deep-ultraviolet (DUV) lithography [35, 36, 41], [42], [43]. For example, a 360-nm-thick SiO_2_ layer can be deposited onto a silicon wafer by atomic layer deposition (ALD). Then, a 300 nm-thick Si layer can be deposited on the SiO_2_ layer. The patterned photoresist (PR) layer is coasted as etch mask and the unwanted region of the Si layer is removed by dry plasma etching to form the metasurface. Then, a PR coating on both side of the sample is used as a protective layer for the remaining steps. Then, deep reactive ion etching (DRIE) is used to remove the Si wafer until it reached the SiO_2_ substrate. Finally, clean the wafer and remove the PR layer with oxygen plasma. It has been demonstrated that the highly efficient and precise lithography can be utilized for the mass production of various dielectric metasurfaces [35].

## 4 Conclusions

In summary, we design a square all-dielectric metasurface and utilize the angle-multiplexed sensing method to realize broadband fingerprint detection of trace-amount analysis and 2D material. The proposed metasurface can provide mid-IR absorbance enhancement of around 10 dB without the need for complex instrumentation. Furthermore, despite a very simple all-dielectric metasurface is designed, it supports sharp Fano resonances governed TD and MD through inducing quasi-BIC with the Q-factor of 818.72, 2.23 × 10^4^, and 1.34 × 10^4^, and sensitivities reach high values up to 1.28, 1.43, and 1.39 μm/RIU, respectively, which achieve superior performance compared to most reported sensors. Therefore, combining the above two sensing methods offers versatile detecting application scenarios and paves the way to label-free and nondestructive trace-amount detecting.
